# An integrated analysis of lncRNA and mRNA expression profiles in the kidneys of mice with lupus nephritis

**DOI:** 10.7717/peerj.10668

**Published:** 2021-02-16

**Authors:** Juan Wang, Xiongfei Wu, Yafang Tu, Jianzhong Dang, Zhitao Cai, Wenjing Liao, Weili Quan, Yaxun Wei

**Affiliations:** 1 Nephrology Department, Renmin Hospital of Wuhan University, Wuhan, Hubei, China; 2 ABLife BioBigData Institute, Wuhan, Hubei, China; 3 Center for Genome Analysis, ABLife Inc., Wuhan, Hubei, China

**Keywords:** Lupus nephritis, RNA sequencing, lncRNAs, Differentially expressed genes, Immune response

## Abstract

Long noncoding RNAs (lncRNAs) are persistently expressed and have been described as potential biomarkers and therapeutic targets in various diseases. However, there is limited information regarding lncRNA expression in the tissue of kidney exhibiting lupus nephritis (LN)a serious complication of systemic lupus erythematosus (SLE). In this study, RNA sequencing (RNA-seq) was performed to characterize the lncRNA and mRNA expression in kidney tissues from LN (MRL/lpr) and control mice. We identified 12,979 novel lncRNAs in mouse. The expression profiles of both mRNAs and lncRNAs were differed significantly between LN and control mice. In particular, there were more upregulated lncRNAs and mRNAs than downregulated ones in the kidney tissues of LN mice. However, GO analysis showed that more downregulated genes were enriched in immune and inflammatory response-associated pathways. KEGG analysis showed that both downregulated and upregulated genes were enriched in a number of pathways, including the SLE pathway, and approximately half of these SLE-associated genes encoded inflammatory factors. Moreover, we observed that 2,181 DElncRNAs may have targeted and regulated the expression of 778 mRNAs in LN kidney tissues. The results of this study showed that 11 DElncRNAs targeted and were co-expressed with six immune and SLE-associated genes. qPCR analysis confirmed that lncRNA Gm20513 positively regulated the expression of the SLE-associated gene H2-Aa. In conclusion, the results of our study demonstrates that lncRNAs influence the progression of LN and provide some cues for further study of lncRNAs in LN. These results regarding the lncRNA-mRNAregulatory network may have important value in LN diagnosis and therapy.

## Introduction

Systemic lupus erythematosus (SLE) is a serious autoimmune disease with a variety of organ manifestations (Kiriakidou & Ching, 2020). The most serious complication affecting most patients with SLE is a kidney manifestation-lupus nephritis (LN) (Davidson, 2016; Davidson & Aranow, 2010). Despite the use of multiple aggressive immunosuppressive therapies, many LN patients still progress to end-stage renal disease (Davidson, 2016). Therefore, it remains important to elucidate the molecular mechanisms mediating the pathogenesis of lupus nephritis.

Omic techniques, including transcriptomic techniques, have become a necessary technique for exploring the underlying molecular mechanisms mediating the pathogenesis of various diseases (Scherer et al., 2017; Soon, Hariharan & Snyder, 2013). In fact, a large number of transcriptomic studies on blood and derived samples from patients have been employed to characterize the gene pathways involved in SLE in an attempt to identify the key pathogenic drivers of the disease (AlFadhli, Ghanem & Nizam, 2015; Grigoriou et al., 2020; Li et al., 2019b; Lood et al., 2010; Panousis et al., 2019; Rai et al., 2016). For example, a recent study demonstrated that the gene expression profile of bone marrow (BM)-derived hematopoietic stem and progenitor cells (HSPCs) from mice with lupus is different from that of healthy controls, which would induce aberrations in immune cells in SLE (Grigoriou et al., 2020). Currently, high-throughput transcriptome sequencing has been employed to study the mechanisms of drug therapy for lupus nephritis, which supports the therapeutic effects of different drugs (Fu et al., 2018; Wang et al., 2019). Transcriptome sequencing of infiltrated T cells in the renal tissue of patients with lupus nephritis elucidated the regulatory mechanisms governing abnormal T cell metabolism and T cell failure (Tilstra et al., 2018). Thus, transcriptomic analysis remains a powerful tool for elucidating the mechanisms mediating the pathogenesis of SLE and LN.

Recently, various noncoding RNAs, including lncRNAs, have been shown to play important roles in the pathogenesis of diseases and therefore represent potential biomarkers and therapeutic targets of those diseases (Esteller, 2011; Matsui & Corey, 2017; Wu et al., 2015a). In fact, several studies have detected the expression profile of lncRNAs in blood and blood- derived plasma or peripheral blood mononuclear cells (PBMCs) from patients with SLE (Geng et al., 2020; Li et al., 2019a; Luo et al., 2018; Wang et al., 2018; Wu et al., 2019; Xu et al., 2020; Ye et al., 2019). For example, lnc-DC and GAS5 were decreased, and linc0597 was overexpressed, in the plasma of patients with SLE, and linc0597, GAS5, and lnc-DC can be used as potential biomarkers for SLE (Wu et al., 2017). Moreover, a number of studies have demonstrated the functional roles of several lncRNAs, including NEAT1, MALAT1, HOXA-AS2, and SLEAR, in SLE according to in vitro loss-of-function and gain-of-function strategies (Fan et al., 2020; Wu et al., 2020; Yang et al., 2017; Zhang et al., 2016). In particular, the lncRNAs THRIL, TUG1 and RP11-2B6.2 were reported to play promotion or inhibition roles in kidney injury in SLE (Cao et al., 2020; Deng et al., 2018; Liao et al., 2019; Xu, Deng & Zhang, 2018). In addition, one study performed RNA-seq to compare the expression levels of lncRNAs in kidney biopsies from patients with LN and patients with kidney tumors (Liao et al., 2019). To the best of our knowledge, no study has systematically compared the expression profile of lncRNAs in kidney tissues from LN patients or animal models with those of healthy controls.

In this study, we hypothesized that both the mRNA and lncRNAs expression profiles of kidney tissues with LN are different from those of a healthy control. To validate our hypothesis, transcriptomic analysis of kidney tissues was performed to investigate the expression of mRNA and lncRNAs in LN and healthy control mice. Our study demonstrates the difference in mRNA and lncRNA expression profiles between LN and control mice. In particular, we constructs lncRNA-mRNA regulatory network in LN. In addition, our study contributes to body of knowledge on this subject and establishes a foundation for further research of lncRNAs in LN.

## Materials & Methods

### Animals

Mice of the strain MRL/MpJ-Fas-lpr (Mrl/lpr), an established model of LN, were used in our study. Control MRL/MpJ mice without the Fas-lpr mutation were used as healthy controls. Specific pathogen-free (SPF)-grade female MRL/lpr and MRL/MpJ mice (weighing 15–16 g and aged 4 weeks) were purchased from SLAC Laboratory Animal Co., Ltd. (Shanghai, China). The purchased animals were maintained in the SPF animal laboratory of Renmin Hospital of Wuhan University. The animals were housed using standard cages in a room with humidity of 50% ± 20%, temperature of 23 °C ± 3 °C, and a 12 h light/12 h dark cycle. The animals had free access to standard laboratory food and water. All animals were fed the same feed. The experiments were conducted after the mice reached the age 18 weeks. Animal studies were approved by the Laboratory Animal Welfare and Ethics Committee of Renmin Hospital of Wuhan University (IACUC Issue No. 20171205).

### Measurements of 24-h UTP, SCR, and anti-dsDNA

According to previous studies, 24-hour urine protein, serum creatinine, and serum anti-dsDNA levels have been employed to estimate the severity of lupus nephritis and therapeutic efficacy of drugs in Mrl/lpr mouse model (Feng et al., 2019; Renner et al., 2015; Wu et al., 2015b).

The urine of mice was collected for 24 h. Urine protein concentration was measured by the Bradford method, and 24-hour urine protein (24-h UTP) was calculated for LN and control mice (*N* = 3 for each group).

Blood samples were collected from the abdominal aorta of LN and control mice (*N* = 3 for each group). Serum was obtained after centrifugation of blood samples for 10 min (3,000 r/min). Serum creatinine concentration (SCR) was measured by the peroxidase method, and serum anti–double-stranded DNA antibody titers (anti-dsDNA) were determined by ELISA kits (Bioyeargene Biotechnology Co., Ltd., Wuhan) according to the manufacturer’s instructions.

### Collection of kidney tissue

Mice were anesthetized by intraperitoneal injection of chloral hydrate (400 mg/kg). The mice were then sacrificed by cervical dislocation and their kidneys were removed (*N* = 3, for each group). The process conformed to the criteria established by the Laboratory Animal Welfare and Ethics Committee of Renmin Hospital of Wuhan University. Fresh kidney tissue was immediately chilled in liquid nitrogen and kept at −80 °C until use.

### RNA extraction, library construction and sequencing

Total RNA was extracted from each kidney tissue sample using TRIzol Reagent (Ambion) according to the manufacturer’s instructions. RQ1 DNase (Promega) was used to eliminate any potential DNA in the total RNA. The quality and concentration of the purified RNA were determined by measuring absorbance at 260 nm/280 nm (A260/A280) in SmartSpec Plus (BioRad). The integrity of the RNA was detected by agarose gel electrophoresis (1.5%).

Next, 10 µg of purified total RNA from each of the samples was purified and concentrated with oligo (dT)-conjugated magnetic beads (Invitrogen) to prepare a directional RNA-seq library. The polyadenylated RNAs were ion fragmented with end repair and 5′ adapter ligation. Primers with known 3′ adapter and random hexamers were used to reverse-transcribe the fragmented RNAs. The resulting cDNAs were subsequently purified and amplified. cDNAs amplification products (200–500 bp) were retained. After quantification, the cDNAs were stored at −80 °C until sequencing.

For high-throughput sequencing, libraries were prepared according to the manufacturer’s protocols. Specifically, 150-bp paired-end sequencing was performed using an Illumina HiSeq 2000 system (ABlife. Inc, Wuhan, China).

### Raw data cleaning and mapping statistics

First, we discarded the raw reads with more than 2-N bases. Adapter were clipped, and low-quality bases were removed. We further discarded short reads (<16 nt) using FASTX-Toolkit (Version 0.0.13, http://hannonlab.cshl.edu/fastx_toolkit/).

Next, TopHat2 was used to map the resulting clean reads to the mouse genome by allowing 2 mismatches (Kim et al., 2013). We discarded the reads that mapped to multiple genomic locations. The unique mapped reads were employed to calculate the FPKM value (FPKM represents reads per kilobase and per million) of each gene.

### Prediction of novel LncRNA in mouse

LncRNA prediction was conducted according to a previous study (Cabili et al., 2011). In brief, Cufflinks (V2.2) was used to assemble the mapped reads with default parameters (Trapnell et al., 2012). Novel transcripts with intergenic and antisense regions were regarded as candidate lncRNAs, which should have less than 1000-bp overlap with known coding genes. The coding potential score (CPS) of candidate lncRNAs should be less than zero, which was calculated by coding potential calculator (CPC) software (Kong et al., 2007). The resulting transcripts that had more than one exon and were longer than 200 bases were defined as lncRNAs. After the antisense reads were discarded, the FPKM value of each lncRNA gene were recalculated.

### Differentially expressed mRNA and lncRNA Genes (DEGs and DElncRNAs)

In this analysis, edgeR was used to identify DEGs and DElcnRNAs according to the FPKM value of each gene in each sample (Robinson, McCarthy & Smyth, 2010). DEGs and DElncRNAs were defined according to the criteria of 2-fold changes and *p*-values <0.05.

### Coexpression network analysis

According to a previous study (Liu et al., 2017), we constructed a coexpression network between lncRNAs and mRNAs. In brief, the correlation coefficients and *p*-values were obtained for each mRNA-lncRNA pair based on the expression of each mRNA and lncRNA. A given threshold (absolute correlation coefficients no less than 0.7 and *p*-values less than 0.01) was utilized to filter the results. The filtered gene pairs form the expression network.

### GO and KEGG analysis

Gene Ontology (GO) and KEGG pathway analyses were used to predict the function of DEGs, which were identified using the KOBAS 2.0 server (Xie et al., 2011). The enrichment of genes in each term or pathway was determined by the hypergeometric test and Benjamini–Hochberg FDR at corrected *p*-value <0.05.

### RT-qPCR validation of DElncRNAs and DEGs

Quantitative real-time PCR (RT-qPCR) was performed to validate the RNA-seq data in this study. We selected some differentially expressed genes and lncRNAs with relatively high and reproducible expression for three biological replicates of each group for qPCR verification. The expression of some selected DEGs and DElncRNAs was quantified by RT-qPCR by using GAPDH as a reference. RT-qPCR was conducted on the RNA samples that were the same as those employed for RNA-seq. The RT-qPCR conditions were as follows: denaturing at 95 °C for 10 min, 40 cycles of denaturing at 95 °C for 15 s, and annealing and extension at 60 °C for 1 min. RT-qPCR of each sample employed three technical replications. The primers for each gene for RT-qPCR are listed in Table S1.

### Statistical analysis

An unpaired two-tailed *t*-test was performed to compare the pathological evaluation and RT-qPCR data from two different groups. SPSS software for Version 19 (IBM, USA) was utilized to perform the statistical test. A significant difference was set at probability (*P*) values less than 0.05 (*p* < 0.05). Each group had at least three biological replicates (n ≥ 3). The data are presented as the mean ± standard deviation (SD).

## Results

### Detection of key physiological indices demonstrated that the lupus nephritis model was established

**Figure 1:**
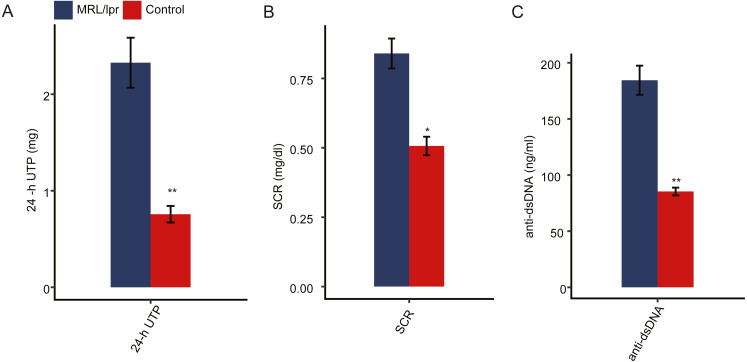
Pathologic characteristics of the lupus nephritis mouse model. (A) Quantitative analysis of 24-hour urinary total protein (24-h UTP). (B) Quantitative analysis of serum creatinine concentration (SCR). (C) Quantitative analysis of serum anti-double-stranded DNA antibody titers (anti-ds-DNA). *N* = 3 for each group. Data are presented as the mean ±  standard deviation (SD). Student’s *t*-test was performed to compare LN mice and controls with the significance set at a *P* value of less than 0.05. *P < 0.05, **P < 0.01.

To ensure the establishment of the LN mouse model, several biochemical indicators of LN mice were detected and compared to those of control mice. Proteinuria usually occurs after 8 weeks of age in MRL/lpr mice. We first measured the 24-hour urinary total protein (24-h UTP) of both LN and control mice. The results showed that the 24-h UTP was significantly higher in LN mice than in control mice (Fig. 1A). Serum creatinine concentration (SCR) and serum anti-double-stranded DNA antibody titers (anti-dsDNA) were also indications of LN. Compared with control mice, SCR was significantly elevated in LN mice (Fig. 1B). In addition, LN mice had a higher concentration of anti-dsDNA in serum than control mice (Fig. 1C). Taken together, these results indicted a lupus nephritis mouse model was established.

### Both the mRNA and lncRNA expression profiles of kidney tissue differed between LN and control mice

**Figure 2:**
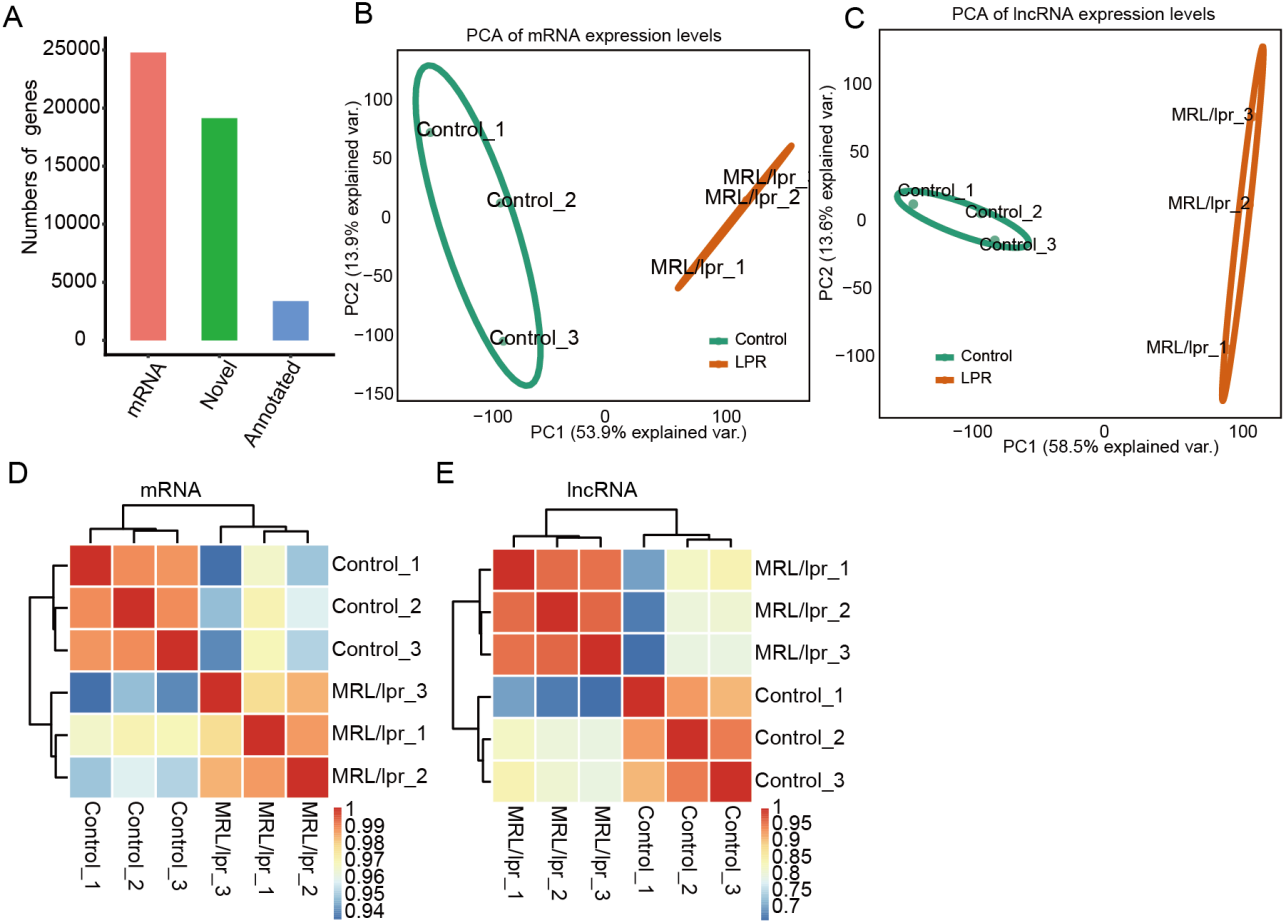
Gene expression profile of kidney tissue from LN and control mice. (A) Number mRNA and lncRNA genes expressed in at least one sample (with FPKM > 0). (B and C) Principal component analysis (PCA) of six distinct samples according to the expression levels of the mRNA (B) and lncRNA (C) genes. The samples were clustered by LN mice and controls. (D and E) Heatmap of the correlation coefficient of six samples according to the expression level of the mRNA (D) and lncRNA (E).

To elucidate the molecular mechanisms governing lncRNAs that may account for lupus nephritis, we profiled the transcriptomes of kidney tissue from LN and control mice (*N* = 3 in each group). In total, six transcriptome data sets were obtained, with each composing an average of approximately 80 million PE-end reads for analysis (Table S2). Next, we aligned the clean reads to the reference sequence of mice with TopHat2 with two mismatches to detect and characterize the expression patterns of annotated genes. In addition, Cufflinks was used to perform *ab initio* transcript assembly and reconstructed transcripts, and 19,327 novel lncRNA genes were identified after a series of filtering steps. In total, there were 28,173 mRNA genes, and 22,504 lncRNA genes were expressed in at least one sample (with FPKM >  0) (Fig. 2A). These expressed mRNA and lncRNA genes were used to characterize the gene expression profile of LN and control mice.

Principal component analysis (PCA) was performed to explore the temporal expression patterns associated with all mRNAs and lncRNAs in our datasets. Both mRNA and lncRNA expression levels could be distinguished between the LN and control samples (Fig. 2B and 2C). Pearson correlation tests for all RNA-seq sample pairs were performed and indicated similar outcomes for mRNA expression and lncRNA expression (Fig. 2E and  2D). These results indicated that the expression profiles, including mRNAs and lncRNAs, of the kidney tissues of LN mice were significantly different from those of the control mice.

### There were more upregulated lncRNAs and mRNAs than downregulated lncRNAs and mRNAs in the kidney tissue of LN mice

**Figure 3:**
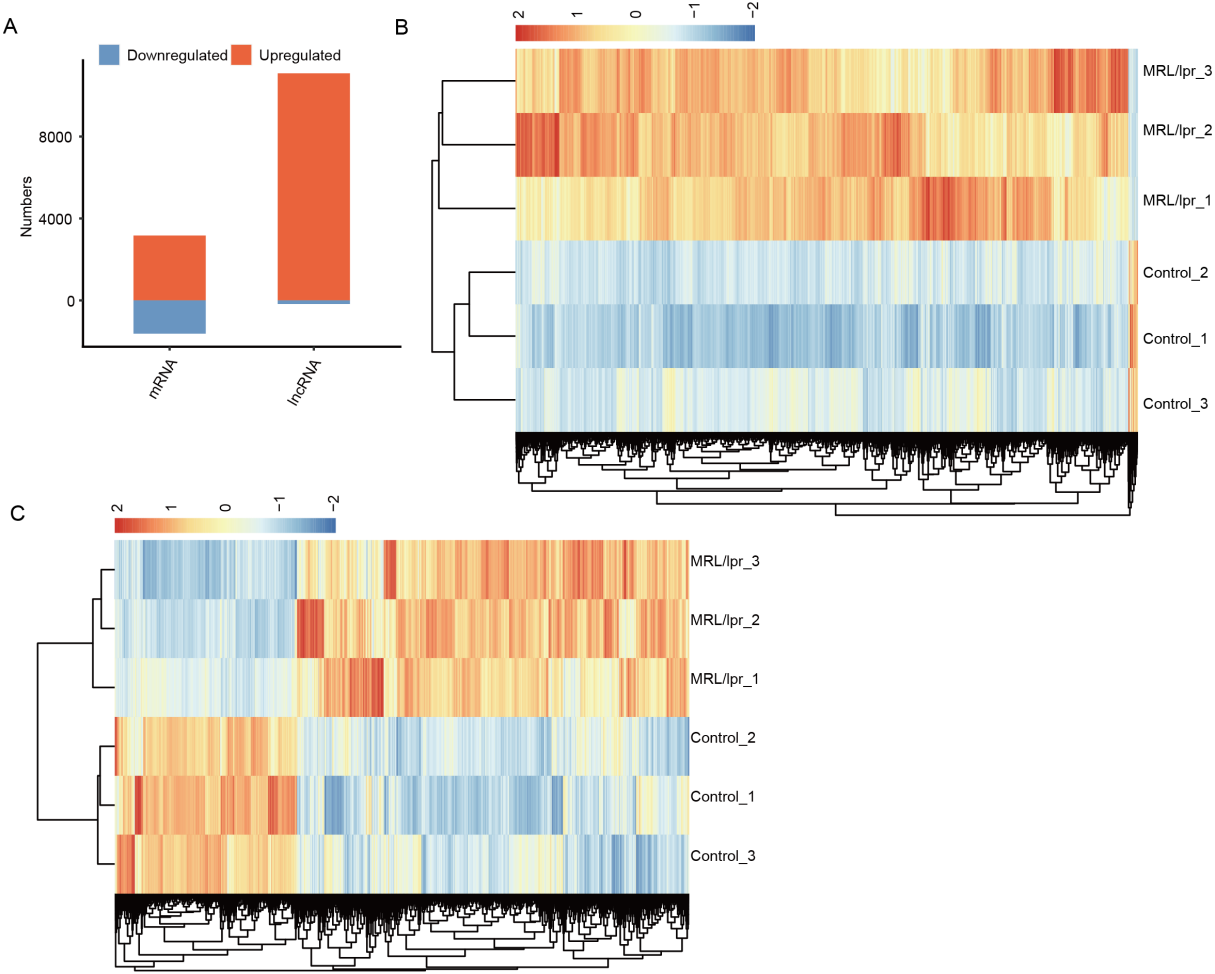
Identification of the differentially expressed mRNAs between the two groups. (A) Distribution of the number of upregulated and downregulated mRNAs and lncRNAs between LN and control mice. (B) Hierarchical clustering and heatmap of six samples based on all differentially expressed lncRNAs. (C) Hierarchical clustering heatmap of all samples based on all the differentially expressed mRNAs.

To explore the difference in mRNA and lncRNA expression profiles, edgeR was performed to identify the differential expression of mRNAs (DEGs) and lncRNAs (DElncRNAs) (≥2-fold up or down, FDR < 0.05) between LNs and controls. In total, there were 11,258 DElncRNAs and 5,266 DEGs (Fig. 3A). In particular, compared to controls, we identified 11,084 upregulated lncRNAs but 174 downregulated lncRNAs in LN (Fig. 3A). Notably, we also identified more unregulated mRNAs than downregulated mRNAs in mice (Fig. 3A).

**Figure 4:**
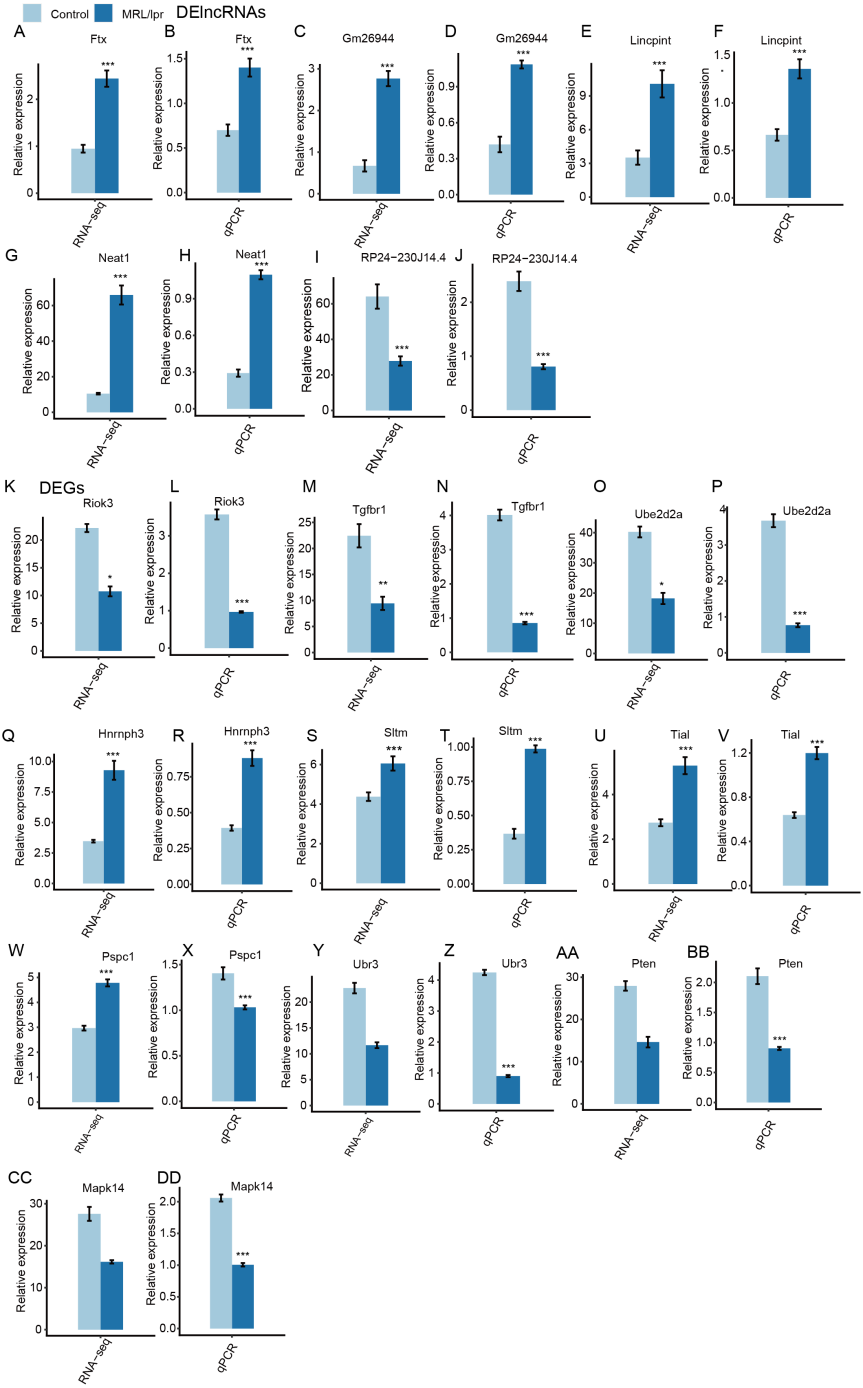
RT-qPCR validation of DEGs and DElncRNAs. Relative expression levels of DElncRNAs (A–J) and DEGs (K–DD) by RNA-seq (FPKM) and RT-qPCR. Data are presented as the mean ±  standard deviation (SD). Student’s *t*-test was performed to compare LN and control mice with the significance set at a *P* value of less than 0.05. *P < 0.05, **P < 0.01.

Next, heatmaps were plotted based on the normalized FPKM values of DElncRNAs. Hierarchical cluster analysis showed a clear separation of LN and control samples with a high consistency for the three replicate data sets (Fig. 3B). These results were in keeping with the resutls obtained for the DEGs (Fig. 3C). Finally, the expression of five lncRNA and ten mRNA was validated by qPCR (Fig. 4). All five lncRNAs and nine out of ten mRNAs showed consistency for qPCR and RNA-seq. These results indicated that the expression at the transcript level of a larger number of mRNAs and lncRNAs was affected in LN kidney tissues in mice.

### Downregulated DEGs but not upregulated DEGs were significantly enriched in immune-and SLE-associated pathways in the kidneys of LN mice

**Figure 5:**
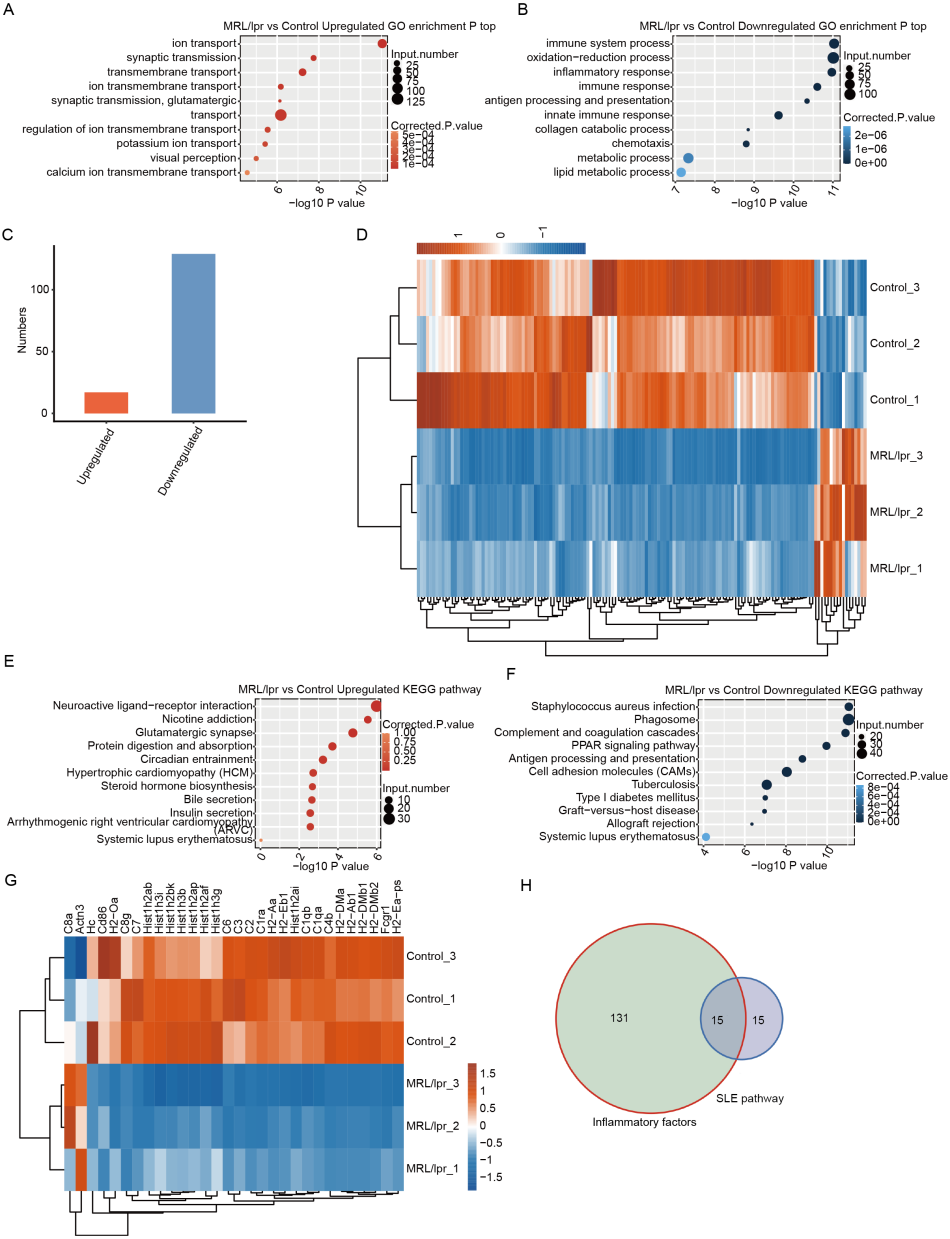
Functional analysis of differentially expressed mRNA genes. (A and B) The top 10 GO analysis terms for upregulated (A) and downregulated (B) DEGs in LN mice. (C) The number of upregulated and downregulated immune and inflammatory genes in LN mice. (D) Hierarchical clustering and heatmap of six samples based on all the differentially expressed immune and inflammatory genes. (E and F) The KEGG pathway for upregulated (E) and downregulated (F) DEGs in LN mice. (G) Hierarchical clustering and heatmap of six samples based on all differentially expressed SLE-associated genes. (H) Overlap of the differentially expressed inflammatory genes and SLE-associated genes.

GO analysis was performed to demonstrate the potential function of all DEGs. There were 625 upregulated and 1,045 downregulated genes that were annotated with GO biological process terms. The upregulated DEGs were enriched in 180 GO terms, and the downregulated DEGs were enriched in 358 GO terms (Tables S3 and S4). Notably, the upregulated DEGs were enriched in terms related to transport and transmission (Fig. 5A). However, the downregulated DEGs were enriched in such terms as immune response and inflammatory response (Fig. 5B). Although the upregulated DEGs were also enriched in immune-and inflammatory-associated pathways, the gene number was significantly lower than that of downregulated genes (Fig. 5C). Next, we merged genes from the four immune-and inflammatory-associated pathways, including “immune system process”, “inflammatory response”, “immune response”, and “innate immune response”, which resulted in 129 downregulated genes and 17 upregulated genes (Table S5). We plotted a heatmap of these genes, which showed that the expression of these genes differed between the LN and control samples (Fig. 5D).

To further demonstrate the functional roles of these DEGs, KEGG pathway analysis was performed for the DEGs. The results showed that upregulated genes were enriched in neuroactive ligand–receptor interactions and glutamatergic synapses (Fig. 5E). The downregulated genes were enriched in such pathways as the response to *Staphylococcus aureus* infection and antigen processing and presentation (Fig. 5F). Notably, both downregulated and upregulated genes were enriched in systemic lupus erythematosus (Fig. 5E and 5F). However, there were 28 downregulated genes associated with systemic lupus erythematosus, but only 2 upregulated genes. A heatmap of these genes could separate the LN and control samples (Fig. 5G). In addition, approximately half of these SLE-associated genes were inflammatory factors (Fig. 5H, Table S6). These results showed that the expression of many immune and inflammatory factors was repressed in kidney tissue with LN.

### LncRNAs may regulate the expression of immune and SLE-associated genes in the kidneys of LN mice

**Figure 6:**
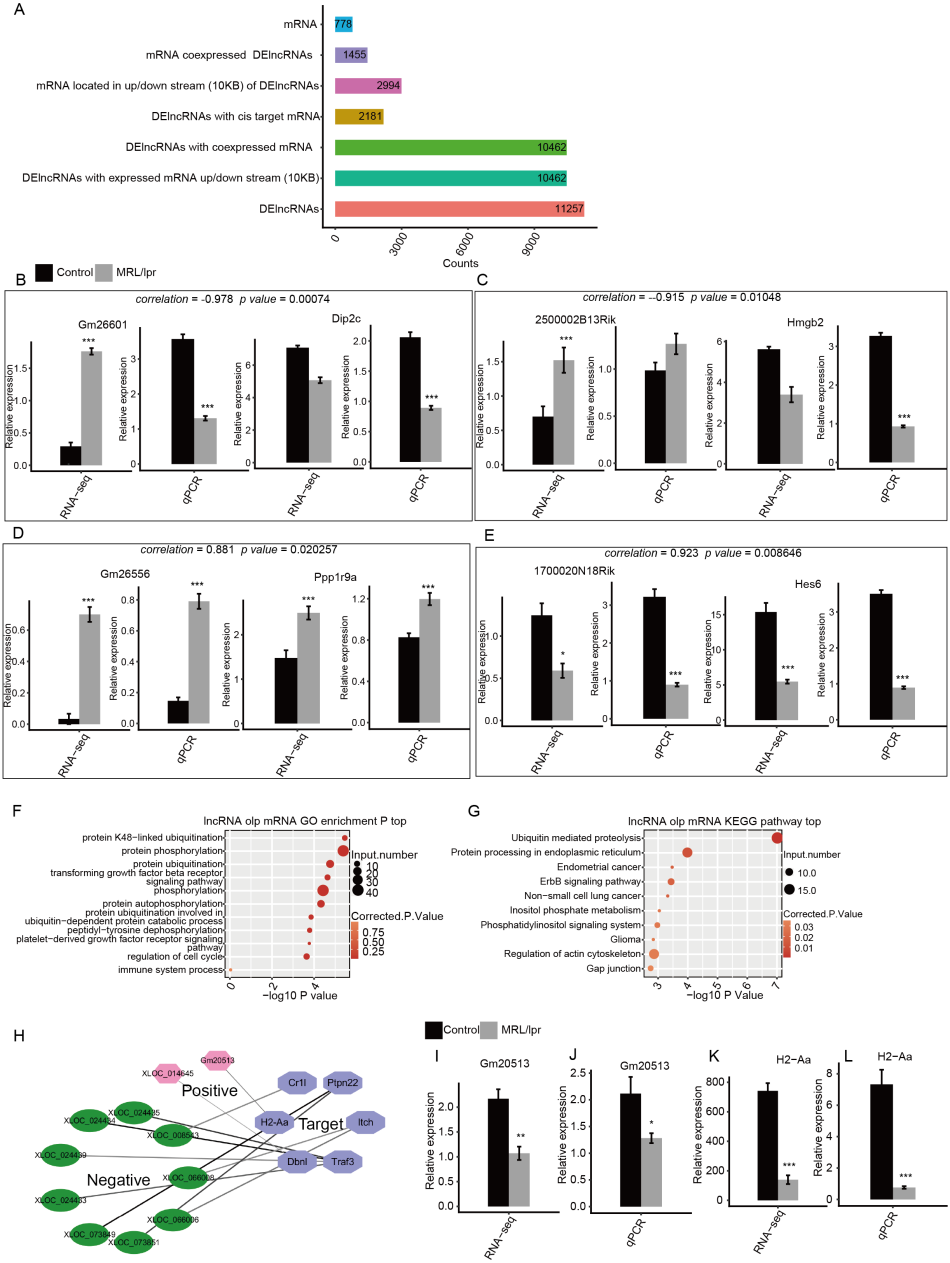
Functional analysis of differentially expressed lncRNA genes. (A) Number of DElncRNAs and their cis-target mRNA genes. (B–E) RT-qPCR validation of the expression levels of four DElncRNAs and their cis-target mRNAs. (F) The top 10 GO analysis terms for DElncRNA cis-target mRNA genes. (G) The KEGG pathway for DElncRNA cis-target mRNA genes. (H) Coexpression network between DElncRNAs and six cis-target SLE and immune genes. (I–L) RT-qPCR validation of the expression levels of the DElncRNA Gm20513 and its cis-target SLE gene (H2-Aa). RNA-seq (FPKM) and RT-qPCR are presented. Data are presented as the mean ±  standard deviation (SD). Student’s *t*-test was performed to compare LN and control mice with the significance set at a *P* value of less than 0.05. * *P* < 0.05, ** *P* < 0.01, *** *P* < 0.01.

To determine the function of these DElncRNAs, we first identified the DElncRNAs with expressed mRNAs located within 10 kb upstream or downstream. There were 10,462 of 11,257 DElncRNAs with 2994 expressed mRNA genes. Next, the Pearson correlation coefficient for the expression of the DElncRNAs and mRNAs was calculated. DElncRNAs and mRNAs were considered to be coexpressed when the absolute value of the correlation coefficient was more than 0.6 with *P* -values <0.05. We identified 7,225,223 coexpressed pairs by 104,62 DElncRNA and 1,455 mRNA. Finally, we identified 778 mRNAs that were located within 10kb upstream or downstream of 2,181 coexpressed DElncRNAs. These mRNAs were considered to be the potential targets of the DElncRNAs (Fig. 6A). The expression of two positive and two negative pairs of DElncRNAs and mRNAs was validated by qPCR (Fig. 6B–E). This result was observed by both qPCR and RNA-seq.

Next, GO analysis was performed to annotate the function of DElncRNA-target mRNAs. The results showed that these genes were enriched in such pathways as protein ubiquitination, protein phosphorylation and the transforming growth factor beta receptor signaling pathway (Fig. 6F). In particular, these genes were also enriched in the immune system process pathway, which includes six genes (Fig. 6F). We then annotated these DElncRNA-target mRNA genes by KEGG pathway analysis. The results showed that these genes were enriched in pathways including ubiquitin-mediated proteolysis and phosphatidylinositol signaling system (Fig. 6G). Notably, one gene (H2-Aa) was annotated with systemic lupus erythematosus according to KEGG analysis (Fig. 6G). Next, we plotted the coexpression network of these SLE and immune genes and DElncRNAs. The results showed that 11 DElncRNAs were coexpressed with these genes and may have targeted these genes (Fig. 6H). qPCR confirmed that lncRNA Gm20513 positively regulated the expression of the SLE-associated gene H2-Aa in kidney tissue (Fig. 6I–L). The qPCR results were consistent with those obtained by RNA-seq. All these results indicated that some lncRNAs may regulate the expression of immune and SLE-associated genes in the kidneys of LN mice.

## Discussion

SLE is a systemic disease that can affect various organs, and there is a high incidence of kidney damage, which is referred to as LN (Davidson & Aranow, 2010). This kidney damage is a considerable cause of mortality and disability in patients with SLE and includes glomerular, tubular, renal interstitial and blood vessel destruction (Wu et al., 2017). In this study, we sought to elucidate the differential molecular signaling pathways involving lncRNAs that may account for increased disease in a mouse model of LN, MRL/lpr mice. Comparison of transcriptomic profiles showed that DEGs were involved in immune, inflammatory response and systemic lupus erythematosus pathways. Pathway analysis showed that DElncRNAs also targeted and regulated the expression of immune and SLE-associated genes, suggesting a potential role played by lncRNAs in immune disorders and kidney injury in LN mice.

In fact, many studies have explored the role of aberrant expression of mRNA, miRNA and lncRNA in blood or blood-derived cells from patients with SLE (Geng et al., 2020; Li et al., 2019a; Luo et al., 2018; Wu et al., 2019; Xu et al., 2020; Ye et al., 2019). To the best of our knowledge, only one study has characterized the differential expression of lncRNAs in kidney tissue between patients with SLE and patients with kidney tumors (paracarcinoma tissues) (Liao et al., 2019). In this study, we examined the profiles of mRNA and lncRNA expression in kidney tissue of LN and normal control mice. Notably, LN and control mice showed differential expression profiles of both mRNAs and lncRNAs in kidney tissues. This result is consistent with the findings of previous studies showing that the expression profiles of mRNAs and lncRNAs in blood or derived cell samples could distinguish SLE and healthy controls (Geng et al., 2020; Luo et al., 2018). For example, our results showed a 6-fold upregulation of lncRNA Neat1 in LN mice. It has been shown that Neat1 functions as a novel inflammatory regulator acting through the MAPK pathway in human lupus (Zhang et al., 2016). Notably, our study showed that both the numbers of upregulated lncRNAs and mRNAs were greater than the number of downregulated lncRNAs and mRNAs, especially lncRNAs. In addition, we identified a large number of novel lncRNAs in the kidneys of mice, and most of these lncRNAs were upregulated. Thus, further research is warranted to verify the reliability of these lncRNAs in the kidney tissue of LN mice.

The immune system is strongly associated with LN (Davidson, 2016; Davidson & Aranow, 2010). In our study, both downregulated and upregulated mRNA genes were enriched in immune response and inflammatory response, suggesting that deregulated mRNAs may play a crucial role in immune regulation in LN. KEGG pathway analysis revealed that some DEGs could contribute to the pathogenesis of SLE. This finding further suggests that the dysregulated activation of immune system function is strongly correlated with LN. In fact, a study showed that SLE induces inflammation by increasing the expression level of the lncRNA TUG1 in mice (Xu, Deng & Zhang, 2018). It is important to determine the potential expression relationship between protein-coding genes and lncRNAs by constructing a lncRNA-mRNA coexpression network (Liu et al., 2017). This approach has been demonstrated to be a useful means deducing the potential function of lncRNAs by examining them in close relation to mRNAs whose functions have already been annotated in SLE (Joslyn et al., 2018; Luo et al., 2018; Wang et al., 2018; Yang et al., 2017). In our study, we showed that 11 DElncRNAs targeted and c-expressed with six immune and SLE-associated genes. For example, lncRNA Gm20513 positively regulates the expression of the SLE-associated gene H2-Aa. H2-Aa is one of the major histocompatibility complex class II molecules (Kaufman et al., 1984), is expressed by intrinsic renal cells and is required for crescentic glomerulonephritis (Li et al., 1998). It is possible that these specific coexpressed mRNA-lncRNA networks are involved in the mechanism underlying the pathogenesis of LN. In fact, a previous study showed that linc00513 was a novel positive regulator of the type I interferon pathway by promoting the phosphorylation of the transcription factors STAT1 and STAT2 in SLE patients (Xue et al., 2018). Long noncoding RNA TUG1 protects renal tubular epithelial cells against injury induced by lipopolysaccharide by regulating microRNA-223 (Xu, Deng & Zhang, 2018). LncRNA THRIL contributes to lipopolysaccharide-induced HK-2 cell injury by sponging miR-34a (Deng et al., 2018). We surmise that lncRNAs sponge microRNAs or interact with transcription factors to regulate the expression of downstream target genes. It would be meaningful to further verify whether Gm20513 regulates the expression of H2-Aa via knockdown or overexpression, and to explore the molecular mechanisms underlying LN in kidney tissues.

## Conclusions

This study presented a comprehensive expression profile of lncRNAs and mRNAs in the kidney tissues of mice with LN. Our study demonstrated that both lncRNAs and mRNAs exhibited different expression profiles in LNs compared with healthy controls. These results help to elucidate the possible mechanisms involving lncRNAs and mRNAs, which are implicated in the development and pathogenesis of LN. Thus, the results of our study establish a foundation for future studies seeking to elucidate the underlying mechanisms of LN via a signal transduction cascade network involving lncRNAs.

##  Supplemental Information

10.7717/peerj.10668/supp-1 Supplemental Information 1 Primers that were used for qPCR in DEGs and DELncRNAs

10.7717/peerj.10668/supp-2 Supplemental Information 2 Summary of sample names, description, the RNA-seq sequencing information and mapping results in each sample

10.7717/peerj.10668/supp-3 Supplemental Information 3 GO biology process terms of upregulated genes in kidney of LN mouse

10.7717/peerj.10668/supp-4 Supplemental Information 4 GO biology process terms of downregulated genes in kidney of LN mouse

10.7717/peerj.10668/supp-5 Supplemental Information 5 List of 129 downregulated genes and 17 upregulated genes enriched in immune- and inflammatory- assocaiated pathways (related to Fig. 5C)

10.7717/peerj.10668/supp-6 Supplemental Information 6 List of inflammatory factors, SLE-associated genes and overlapped genes (related to Fig. 5F)

10.7717/peerj.10668/supp-7 Supplemental Information 7 Urine protein content, serum creatinine concentration, serum anti-double-stranded DNA antibody titers, & RT-qPCR raw data

## Additional Information and Declarations

### Data Availability

The following information was supplied regarding data availability: Raw data are available in the NCBI Gene Expression Omnibus: GSE153547. Additional data are available as a Supplemental File.
